# Parents' knowledge and behaviour concerning sunning their babies; a cross-sectional, descriptive study

**DOI:** 10.1186/1471-2431-6-27

**Published:** 2006-10-31

**Authors:** Nihal Aladag, Tuncay M Filiz, Pinar Topsever, Suleyman Gorpelioglu

**Affiliations:** 1 Department of Family Medicine, Kocaeli University Faculty of Medicine, Kocaeli, 41000 Turkey

## Abstract

**Background:**

For centuries, sunlight has been used for therapeutic purposes. Parents still sun their infants to treat neonatal jaundice, nappy rash or mostly to supply vitamin D for bone development as a consequence of health beliefs. In this study we aimed to assess knowledge and behaviour of parents about benefits of sunlight and sun protection.

**Methods:**

In this study, parents attending to governmental primary healthcare units for their children's routine vaccinations, upon their informed consent, were consecutively enrolled during one month. Data were collected by a semi-structured questionnaire.

**Results:**

The mean age of 118 enrolled parents and their babies were 27.9 ± 6.5 years and 8.3 ± 5.8 months, respectively. Most of the participants were mothers (93.2%), housewives (81.4%) with an educational level of ≥6 years (71.2%). Sunlight was considered beneficial for bone development (86.4%), diaper rash (5.9%) and neonatal jaundice (12.7%). In case of neonatal jaundice 72.0% of the participants reported that they would consult a physician. Most of the participants (82.2%) were sunning their babies outdoors. Nearly half (49.7%) of them got this information from medical staff. Fifty two percent of the parents were sunning their babies before 10–11 a.m. and/or after 3 p.m. Only 13.6% of parents reported using sunscreen for their babies, and the majority of them were using sun protecting factor ≥ 15. One forth of the sunscreen users was using sunscreen according to their physicians' advice.

**Conclusion:**

Most of the participants were aware of the benefits of sunlight; especially for bone development. However they were displaying inappropriate behaviour while sunning their babies for health reasons. More education should be given to parents about the danger of sunlight at primary health care units while advising to sun their babies, if any.

## Background

For centuries, sunlight has been used for therapeutic purposes (heliotherapy), which dates back to ancient Rome and Greece [1]. In the second half of the 19^th ^century, it was realized that sunshine could have bactericidal effect as well as a therapeutic role in rickets [2-5]. In 1958, sunlight was first used for neonatal jaundice [2,6,7]. Placing a child in a room where sunlight enters through window panes (not in direct sunlight) for 10 minutes twice a day was often used to help treat mild neonatal jaundice [8].

As the skin cancer rates began to climb in the 1940s and turned into an epidemic by the 1970s, scientists realized the damaging effects of sunlight [4]. Currently, the American Academy of Dermatologists advises to obtain vitamin D through diet or oral supplements and intentional sun exposure for this purpose is not recommended [9]. The therapy of choice for neonatal jaundice in infants is phototherapy [10].

In Turkey, melanoma due to sunlight is underreported. According to the Turkey Health Report 2003, cancer is the second cause of death and, among others, skin cancer is one of the frequently reported forms of cancer. However, it is stated that the cancer reports in Turkey are poor and data are incomplete and of poor quality, thus leading to underreported figures for different types of cancer, including skin cancers [11]. Another source in 1999 conveyed that 5.74% of all reported malignancies were skin cancers [12]. It was reported that among top ten cancers 5.0% and 6.9% were skin cancers in men and women, respectively. However, the incidence of melanoma among these skin cancers was unclear [13-15]. In the European Health Report 2005 the average deaths per 100 000 population from melanoma of the skin was reported as "not available" for Turkey [16].

It has been suggested that a person's lifetime sun exposure mostly occurs during childhood [14]. In many articles, it has been demonstrated that exposure to sunlight as an infant increases the risk of the development of melanoma and other skin cancers later in life [3,17-22]. It was also suggested that in determining the risk of skin cancer, age at which direct sunlight exposure is initiated is more important than the total sunlight exposure over a lifetime [23]. Therefore, sun protection for children is of high importance. Recommendations for sun protection are to wear protective clothing, apply sunscreen with a sun protection factor (SPF) at least 15, apply sunscreen 15 to 20 minutes before going outside, reapply sunscreen every 2 hours or immediately after swimming and to avoid UV exposure between the peak exposure hours of 10_AM _and 4_PM _[24-27].

In a previous study mothers cited health benefits as the reason for exposing their children to the sun [17]. Parents still sun their infants to treat neonatal jaundice, nappy rash or mostly to supply vitamin D for bone development as a consequence of health beliefs [1].

Vitamin D deficiency and nutritional rickets are still health problems in developing countries. Despite Turkey being in a geographical location with abundant sun light exposure, Vitamin D-deficiency continues to be a major health problem. Yearly incidence rates of Vitamin D-deficiency reported in Turkey vary from 1.67%–19%, changing according to study, time span and region. The incidence of nutritional rickets in Turkey for babies smaller than 3 years is 6%. Thus, sunning for bone development is still being advised [28,29]. Since many parents in Turkey also still expose their children to sunlight with the understanding that it is beneficial, we wanted to assess knowledge and behaviour of parents about sunlight exposure and sun protection.

## Methods

This cross-sectional, descriptive survey was conducted in Degirmendere, a small town (population 22.086) in the province of Kocaeli, in Northwest Turkey. Kocaeli is located between 29° 22'–30° 21' eastern longitude and 40° 31 '–41° 13' north latitude, where the climate is moderate with an average of two months of abundant sunshine through the year (July-August) [30]. The UV index for Kocaeli is 8, which is defined as "high" [31,32]. Maximum temperature from mid-June to mid-August is approximately 30–35°C. At this time of the year the bright sunshine duration is approximately eight hours per day. The population of Degirmendere is Caucasian and skin type ranges between 2–4 according to Fitzpatrick's skin types [33].

The target population consisted of all parents attending to governmental primary healthcare units for their children's routine vaccination in a period of one month. A semi-structured questionnaire was used for collecting data. The questionnaire consisted of 14 items investigating parents' knowledge, attitudes and behaviour about sunlight exposure and sun protection of their baby, as well as socio-demographic data. The interview included both, open-ended and set-response questions (see Additional file 1). The results of a pilot survey of 20 interviews were utilized to make changes for better understanding of the questions and to eliminate redundancy.

The study was performed in November 2003. Parents who brought their children for vaccination were consecutively approached by one of the researchers for their informed consent to be enrolled in the study. None of the parents refused taking part in the study. The questionnaires were completed by the participants just after their children received the vaccination(s). Furthermore, the participants were dichotomized according to their education level as ≤5 years of education and ≥6 years of education.

In this study, SPSS 11.0 for windows was used for coding and data analysis. Data were analyzed using descriptive methods and presented as % or mean +/- standard deviation (SD).

## Results

About 655 children were expected to be vaccinated in the year 2003. A total of 118 parents (18% of the target population) were enrolled in this study.

### General characteristics of the study population

The mean ages of parents and their babies were 27.9 ± 6.5 years and 8.3 ± 5.8 months (range 1–23 months, median 6), respectively. The majority of the participants were mothers (n = 110, 93.2%), and most of them were housewives (n = 96, 81.4%). More than two thirds of participants (n = 84, 71.2%) had a school education level of ≥6 years.

### Parent's health beliefs about benefits of sunlight and attitudes towards jaundice

Sunlight was considered beneficial for diaper rash by seven (5.9%) participants. One of them cited her mother as the information source whereas, the others claimed to be acting upon own experience. Most of the participants (n = 102, 86.4%) reported that sunlight was good for their baby's bone development and one third of them had gathered this information from health care providers. When asked about jaundice, 15 participants (12.7%) claimed that sunlight was good for jaundice, and five of them referred to a health care provider as the source of information (Table 1, 2).

**Table 1:** Knowledge about sunlight

	Good for nappy rash n (%)*	Good for jaundice n (%)*	Good for bone development n (%)*
Yes	7 (6.5)	15 (14.0)	*102 (87.9)*
No	8 (7.5)	7 (6.5)	*0*
No idea	92 (85.9)	85 (79.4)	*14 (12.1)*
Total **	*107 (100)*	*107 (100)*	*116 (100)*

*valid percent

**non-respondent: 11, 11 and 2, respectively

**Table 2:** Sources of information for the benefits of sunlight

		Bone development n (%)*	Neonatal jaundice n (%)*	Nappy rash n (%)*
Medical staff	Doctor	18 (23.7)	4 (57.1)	-
	Nurse	11 (14.5)	1 (14.3)	-
Media		10 (13.2)	-	-
Neighbours		13 (17.1)	-	-
Parents		11 (14.5)	1 (14.3)	*1 (50.0)*
Self experience (previous baby)		13 (17.1)	1 (14.3)	*1 (50.0)*
Total**		*76 (100)*	*7 (100)*	*2 (100)*

*valid percent

**non-respondent: 42, 111 and 116, respectively

To the question "what to do if your baby develops jaundice", 85 (72.0%) participants reported that they would go to see a physician whereas, one participant told that she would give her baby water with sugar. Four (3%) participants told that they would do nothing, and 26 (22%) participants left this question unanswered.

### Behaviour about sun exposure and sun protection

While most of the participants (n = 97, 82.2%) told that they exposed their babies to sun outdoors, nearly one third (35.6%) of mothers reported to expose their babies to sun indoors in front of a window for health reasons (Table 3).

**Table 3:** Behaviours about sunning and sun protection

	Sunning outside n (%)*	Sunning behind the window n (%)*	Using sunscreen while sunning n (%)*	
Yes	97 (82.2)	42 (35.9)	16 (14.5)	*2 (1.8) at certain times*
				*5 (4.5) at certain conditions*
No	21 (17.8)	75 (64.1)	*84 (76.4)*	
Total**	118 (100)	*117 (100)*	*110 (100)*	

*valid percent

**non-respondent: 0, 1 and 18, respectively

About half (n = 48, 49.7%) of the mothers who exposed their babies to sun outdoors had gathered this information from medical staff, while one third (n = 29, 29.9%) of them claimed their information source to be their neighbours.

Half of the participants (n = 34, 52%) who had answered (n = 65, 55.1%) the question about the timing of sun exposure reported to display appropriate behaviour (sunning their infant before 10–11 a.m. and/or after 4 p.m.). Fifty three babies (44.9%) were reported to be exposed to sun longer than 15 minutes.

Sixteen (13.6%) mothers reported using sunscreen for their babies before sun exposure. About half of them told that they apply sunscreen only at certain times, e.g. noon time or when they were at the beach (Table 3). Fourteen sunscreen users (11.8%) had been using sun protecting factor ≥ 15. Four (28.6%) of the sunscreen users were using sunscreen according to their physicians' advice, while five (4.2%) of them were using sunscreen based on the information acquired through friends, television and radio.

Only 15 (12.7%) participants replied to the question about when to use sunscreen. Among these answers, more than half (n = 9, 60.0%) were correct (i.e. minimum 30 minutes in advance). Two participants claimed their information source to be physicians, one reported television, and four participants cited their own experience.

## Discussion

The main result of this study was that many parents unintentionally expose their babies to the dangers of sunlight, because of inaccurate information usually due to cultural health beliefs, even when they had been informed by medical/scientific sources.

Although, potential harmful effects of sun exposure in addition to its potential benefits is known for more than half a century, most parents do not seem to have enough information about sun protection and healthy sun exposure. It was shown by various studies that high levels of sun exposure are commonly combined with a low level of sun protection [17].

In our study most of the participants believed that sun exposure is beneficial for their infants, as shown in some other studies [1,17]. In a study conducted in Australia it was reported that nearly half of the participating medical staff recommended sun exposure to treat neonatal jaundice [34]. There is insufficient evidence about the benefit of sunlight for neonatal jaundice [7] and there are no reports/recommendations about the benefit of use of direct sunlight for neonatal jaundice in the medical literature [10]. Nevertheless, especially in developing countries it is believed that exposure to sunlight is beneficial, even considered an alternative phototherapy source by medical staff for the treatment of neonatal jaundice [1,6,35,36]. In this study only a small group of participants thought that sunlight was beneficial for neonatal jaundice. Nonetheless, the parents still preferred consulting a physician in case of jaundice instead of sunning, as shown in another study [1].

In the present study, the great majority of participants thought that it was necessary to expose their baby to sun for appropriate bone development based on the information obtained from medical staff. This rate was higher compared to other studies which had been performed in tropical countries, where there is excess sunshine every season of the year [1,17]. Because Vitamin D-deficiency is prevalent in Turkey, primary health care providers still advise mothers to sun their babies [28].

Only a small proportion of participants believed that sun exposure was necessary for diaper rash, due to their own or their mother's previous experiences, which was similar to the findings of another study [1]. There are no recommendations/or reports about the use of direct sunlight for diaper rash in guidelines [37].

Similar to our results, in a study which was performed in a tropical country, it was shown that only a small proportion of mothers were using sunscreen for their children and among this group, less than one fourth of mothers were applying sunscreen half an hour or more prior to sun exposure and staying more than 15 minutes in the sun [18]. On the other hand, in a study performed in Malta, more than one third of the participants were found to apply sunscreen to their children [19]. Most of the sunscreen users in this study preferred the correct sun protecting factor (SPF 15+), which is consistent with other studies in the literature [18,19].

In our study population we found that the major information source which determines parents' behaviour was either own experience, or experiences of neighbours and friends. We also believe that a possible reason for low rates of sun protection may be the fact that this study was performed in winter and most of the parents had not yet entertained the idea of sun exposure and thus did not feel the need to gather information about sun protection.

Most of the participants told that they exposed their baby to sun outdoors rather than indoors (table 4). Health care providers and neighbours were found to be the major information sources for this behaviour. In the medical literature, it is recommended by an expert group of Turkish paediatricians to instruct/educate mothers to sun their infants at least 20 minutes per day outdoors, stressing that sunning behind the window does not allow the uv-rays stimulating D-vitamin synthesis to pass, and thus, the desired health effect cannot be obtained by doing so [28].

One other finding which was different from the literature [19] was the fact that no one cited school education as an information source for sunning or sun protection of their baby. The main reason for this may be the lack of formal information on sun protection during school education due to possible underreported prevalence of skin cancers in Turkey.

## Conclusion

Our data showed that parents believed that sunlight is good for health, especially for bone development. However they were still displaying inappropriate behaviour about sun exposure of their baby. Therefore, it is critical that parents be informed about the potential benefits and risks of sunlight exposure to their children.

It can be suggested that effects of sunlight and the way of prevention from these harmful effects should become a part of school education programmes. Also, early parental education about how to sun their babies safely should be given especially in primary care.

In some areas, primary health care providers may be focusing on prevention of rickets as rickets is more common than melanoma (or it was reported so) and thus, may be providing one-sided information regarding potential benefits of sun exposure more than potential damage. This may have led to the current behaviour of parents and underestimation of the potential damage from unprotected sun exposure.

Further research should be conducted to assess the knowledge and understand the attitudes about the effects and side effects of sunlight of health care professionals. Large-scale studies are needed for better understanding of parents' behaviours regarding sun exposure.

## Competing interests

The author(s) declare that they have no competing interest

## Authors' contributions

NA: Interviewed the patients, participated in design of the study, did statistical analyses and wrote the manuscript

TMF: Participated in the design of the study and helped to write manuscript

PT: Participated in design of the study and helped to do statistical analyses and to write the manuscript

SG: Reviewed the manuscript for scientific and intellectual content

All authors read and approved the final manuscript

## Pre-publication history

The pre-publication history for this paper can be accessed here:



## Supplementary Material

Additional File 1 Questionnaire form. Click here for file
