# Supplementary material for: Parents' knowledge and behaviour concerning sunning their babies; a cross-sectional, descriptive study
Source: BMC Pediatr. 2006 Oct 31;6:27. doi: 10.1186/1471-2431-6-27 (PMC1634747; doi:10.1186/1471-2431-6-27)
Supplement: Additional File 1 — Questionnaire form. [file 1471-2431-6-27-S1.doc]

1. Who completed the questionnaire?

1. Mother
2. Father
3. Other……

2. Date of birth (yours):

3. Education level:

1. Illiterate,
2. Literate
3. Primary school
4. Secondary school
5. High school
6. University

4. Occupation:

1. Student
2. Employee
3. Labourer
4. House wife
5. Retired
6. Other…….

5. Date of birth of your youngest child:........................................

6. What has to be done in case of neonatal jaundice?........................................

7. Do you sun your baby behind the window indoors? Yes No

8. Do you sun your baby outdoors? Yes No

9. Did anyone recommend you to do so, if you are sunning your baby outdoors?

1. Yes
   1. Physician
   2. Midwife/nurse
   3. Television/radio
   4. Neighbours/elderly people
   5. Other……
2. No

10. At what time of the day do you sun your baby outdoors?

Between……..and…………o’clock.

………minutes.

11. Dou you use sun screen lotion/cream when sunning your baby?

1. Yes…. Who recommended it?...
2. No
3. If we go out between…… and…… o’clock
4. Only at the seaside or at the swimming pool.

12. What is the sun protection factor of the sun screen you prefer for your baby?

1. 8
2. 10
3. 15
4. 20 and over

13. How long do you wait after sun screen application before sunning?

14. Sun is good for…..

|  | Yes | No | No idea | Source of information |
| --- | --- | --- | --- | --- |
| Diaper rash |  |  |  |  |
| Bone development |  |  |  |  |
| Neonatal jaundice |  |  |  |  |
